# Coexistence of Incontinentia Pigmenti and Pituitary Aplasia in a Female Child

**DOI:** 10.7759/cureus.111668

**Published:** 2026-06-28

**Authors:** Marta Valério, Gonçalo Passos Croca, Patrícia Veríssimo, Rita Justo Pereira, Lurdes Lopes

**Affiliations:** 1 Department of Paediatrics, Unidade Local de Saúde da Lezíria, Hospital Distrital de Santarém, Santarém, PRT; 2 Department of Paediatrics, Unidade Local de Saúde do Oeste, Unidade das Caldas da Rainha, Caldas da Rainha, PRT; 3 Department of Paediatric Endocrinology, Unidade Local de Saúde de São José, Hospital Dona Estefânia, Lisbon, PRT

**Keywords:** bloch–sulzberger syndrome, growth-hormone deficiency, ikbkg gene mutation, incontinentia pigmenti, pituitary aplasia

## Abstract

Incontinentia pigmenti (IP) is a rare X-linked dominant neurocutaneous ectodermal disorder. It is caused by IKBKG gene mutations, leading to nuclear factor-kappa B (NF-kB) signaling pathway dysfunction. Skin involvement occurs in almost all patients. IP may also involve teeth, eyes, hair, central nervous system, bone structures, skeletal musculature and immune system. However, endocrine involvement, and specifically the coexistence of IP with complete pituitary aplasia, has not been previously documented in a patient with genetically confirmed IP.

We describe a four-year-old female with genetically confirmed IP referred to Paediatric Endocrinology clinic due to short stature and poor growth velocity. Investigation revealed pituitary aplasia with ectopic neurohypophysis.

The patient initiated hormone replacement therapy with recombinant human growth hormone and hydrocortisone, followed separately by desmopressin, with significant clinical improvement and no exacerbation of cutaneous manifestations. To our knowledge, this is the first documented case of complete pituitary aplasia in a patient with genetically confirmed IP, with a three-year clinical follow-up.

## Introduction

Incontinentia pigmenti (IP), or Bloch-Sulzberger syndrome, is a rare X-linked dominant neurocutaneous disorder. It mainly occurs in females; some males survive due to somatic mosaicism or hypomorphic mutations [1,2].

IP is caused by loss-of-function mutations in the *IKBKG* gene, located at Xq28. This gene encodes the nuclear factor-kappa B (NF-kB) essential modulator, which activates the NF-kB signaling pathway. NF-kB plays a critical role in regulating immune responses, inflammation, and cell survival [1-3]. Beyond its immunological functions, NF-kB signaling is also implicated in the morphogenesis of other ectodermal structures, such as hair follicles [4]. Since the anterior pituitary gland arises from the oral ectoderm through the formation of Rathke’s pouch, and IP results from impaired NF-kB signaling - via mutations in *NEMO/IKBKG* - specifically in ectodermal tissues, a similar disruption of this pathway during Rathke’s pouch development offers a plausible embryological mechanism linking IP to the pituitary aplasia observed in this case. However, as with other ectoderm-derived manifestations of IP, such an effect would be expected to follow the variable expressivity that characterizes this disorder [5].

Clinically, it manifests by characteristic skin lesions progressing through four stages - vesicular, verrucous, hyperpigmented, and hypopigmented - along Blaschko’s lines. Multisystem involvement includes dental, ocular, hair and central nervous system abnormalities [1,6,7]. Structural brain anomalies, such as agenesis of the corpus callosum, polymicrogyria, and gray matter heterotopias, are reported in some patients [8]. Despite this broad multisystem involvement, endocrine manifestations of IP remain poorly characterized, and whether this reflects true rarity or under-recognition is not established. Recognizing pituitary involvement in IP is clinically relevant, as undiagnosed hypopituitarism can lead to growth failure, adrenal crisis, or other preventable complications, all of which are treatable once identified through targeted endocrine screening [9]. Indeed, neither the largest nationwide cohort of IP patients published to date [10] nor the standard clinical genetics reference for this disorder [8] report systematic endocrine assessment as part of their phenotypic characterization, underscoring the current gap in knowledge regarding endocrine outcomes in this disorder.

The latest data estimate a birth prevalence of 2.37 per 100,000 live births [10].

Congenital hypopituitarism is an endocrine disorder resulting in the deficiency of one or more pituitary hormones, often caused by developmental defects such as pituitary stalk interruption syndrome or, more rarely, pituitary aplasia [11].

Pituitary involvement in IP is exceptionally rare, with a single previous report describing a pituitary incidentaloma in a patient with hypothyroidism [12]. To our knowledge, the present case is the first to document complete pituitary aplasia in a patient with genetically confirmed IP.

## Case presentation

We describe a rare case of a four-year-old female patient with IP, referred to the Paediatric Endocrinology Unit due to short stature.

Family history was remarkable for a medically terminated pregnancy at 16 weeks due to fetal hydrops. The etiological study revealed a 46 XY karyotype and an autopsy consistent with a nonspecific polymalformative syndrome.

The patient’s pregnancy was notable for first-trimester gestational diabetes. Serologies were negative. Fetal morphological ultrasound revealed hyperechoic bowel with loop dilation, without other structural abnormalities. She was born at 37 weeks of gestation by spontaneous vaginal delivery. Birth weight was 3620 g (-0.79 SDS), length 42 cm (-2.8 SDS), and head circumference 32 cm (-0.9 SDS), consistent with small for gestational age (SGA).

Newborn physical examination identified subtle craniofacial dysmorphisms - a narrow palate and peculiar facial features. Due to persistent nasal obstruction, on the sixth day, a nasopharyngolaryngoscopy and computed tomography scan confirmed bilateral choanal hypoplasia. After a short course of intranasal corticosteroids, her symptoms improved. Newborn metabolic screening and transfontanellar ultrasound were unremarkable.

On the 11th day, the patient developed cutaneous lesions, initially localized in the perineal region and spreading to the scalp, back and proximal thighs. The eruption began as erythematous patches, evolving into vesicular lesions (stage I) that progressed to linear, infiltrative plaques (stage II, verrucous), followed by hyperpigmented lesions along Blaschko’s lines (stage III), and ultimately leading to scarring lesions and linear alopecia (stage IV, atrophic) (Figure 1). She was treated with topical corticosteroids with good clinical results.

**Figure 1:**
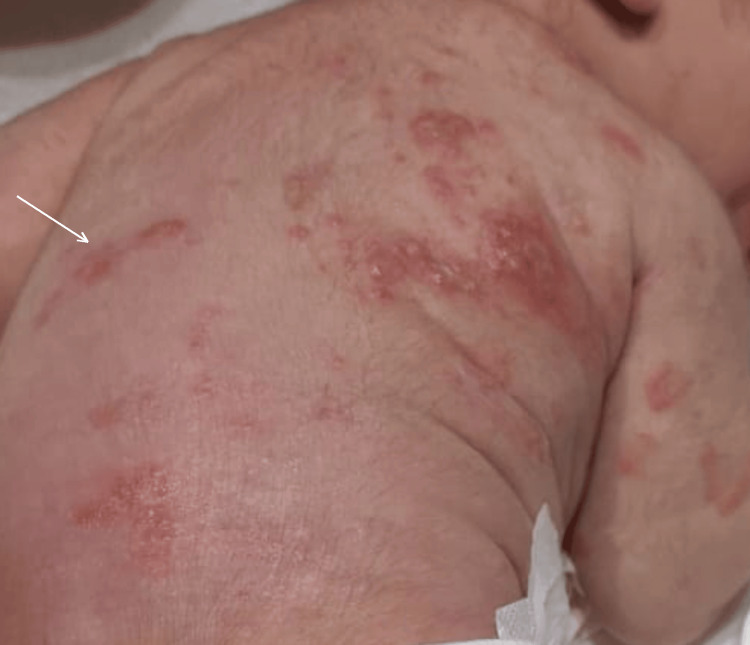
Stage I (vesicular) cutaneous lesions of incontinentia pigmenti on the upper and lower back and shoulder, photographed on day 15 of life, four days after lesion onset on day 11. An isolated linear vesicular lesion progressing along a Blaschko’s line is shown (white arrow). These lesions resolved with topical corticosteroids alone, without further complications.

The onset of the cutaneous lesions prompted follow-up in the dermatology clinic during the first year of life. The clinical suspicion of IP was confirmed by genetic testing, which identified a heterozygous pathogenic variant in the IKBKG gene: c.1178del p.(Pro393Leufs*58). She remained under regular follow-up with dermatology and her family physician. Due to a growth pattern with weight and height consistently below the 3rd percentile for age and sex, she was referred to Paediatric Endocrinology consultation at four years of age, where her weight was 12.5 kg (-2.07 SDS) and her height was 91.7 cm (-2.82 SDS).

Etiological workup showed a suppressed insulin-like growth factor 1 (IGF-1) level of 11.4 ng/mL (reference values 53.0 - 216 ng/mL), suppressed adrenocorticotropic hormone (ACTH) of 4.3 pg/mL (reference values 7.2-63.3 pg/mL), and a low basal cortisol of 2 μg/dL (reference values 2.4-15 μg/dL), with preserved thyroid function (Table 1). Bone age, assessed using the Greulich-Pyle method, was delayed by more than two years. The patient also presented with diurnal enuresis, initially regarded as a nonspecific finding within her neurodevelopmental profile, which was later attributed to central diabetes insipidus once pituitary aplasia was identified on neuroimaging. This sequence illustrates how subtle endocrine deficiencies may initially overlap with, or be mistaken for, neurodevelopmental symptoms in children with IP, particularly in the absence of other clinical red flags directing suspicion toward the endocrine system.

**Table 1:** Endocrine findings at diagnosis (age four years) and after three-year follow-up (age seven years and seven months). The two IGF-1 reference ranges reflect age-specific norms at each timepoint. Suppressed IGF-1 indicated growth hormone deficiency, which normalized with rhGH therapy. Suppressed ACTH and low basal cortisol indicated secondary adrenal insufficiency, which normalized with hydrocortisone. TSH and free T4 remained within the reference range throughout, and no thyroid hormone replacement was required. IGF-1: insulin-like growth factor 1; ACTH: adrenocorticotropic hormone; TSH: thyroid-stimulating hormone; T4: thyroxine; rhGH: recombinant human growth hormone.

Parameters (units)	Patient Values at Diagnosis	Patient Values After Three-Year follow-up	Reference Values
IGF-1 (ng/mL)	11.4	262 (ref. 87.3-324)	53.0-216
ACTH (pg/mL)	4.3	7.4	7.2-63.3
Basal cortisol (μg/dL)	2	7.29	2.4-15
TSH (mUI/L)	1.21	1.32	0.62-4.90
Free T4 (ng/dL)	1.14	1.2	0.96-1.77

At presentation with growth failure, the differential diagnosis included constitutional growth delay, chronic systemic disease, and primary hypothyroidism; the latter was excluded by preserved thyroid function on initial testing. The combination of suppressed IGF-1, ACTH, and basal cortisol, together with structural pituitary aplasia on MRI, was instead consistent with multiple pituitary hormone deficiencies.

Given the clinical suspicion of pituitary dysfunction, brain magnetic resonance imaging was performed, revealing pituitary aplasia with complete absence of the adenohypophysis and neurohypophysis, and possible ectopic foci of pituitary tissue (Figure 2). This pattern is consistent with a developmental disruption of pituitary organogenesis, given the shared ectodermal origin of the anterior pituitary and the tissues primarily affected in IP. Because the anterior pituitary develops from the oral ectoderm via Rathke’s pouch, we propose that any patient with an ectodermal disorder such as IP who presents with poor growth or developmental delay should be promptly screened for congenital hypopituitarism, even in the absence of classic pituitary symptoms. Consequently, the phenotypic presentation of this patient cannot be meaningfully compared against other reported cases, as this is, to our knowledge, the first reported case of this specific coexistence.

**Figure 2:**
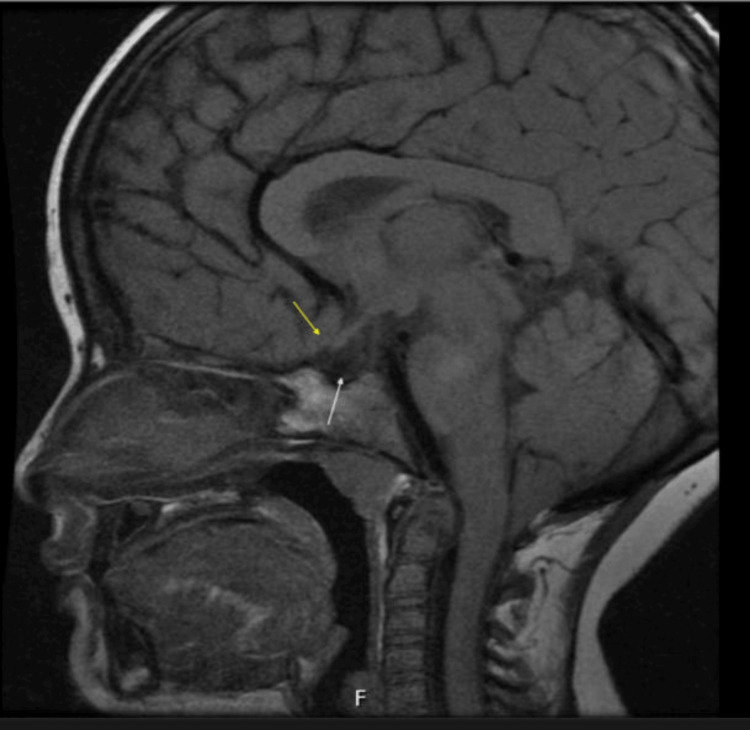
Sagittal T1-weighted magnetic resonance imaging The white arrow indicates an empty sella turcica with absence of a normal anterior pituitary gland, consistent with pituitary aplasia. The yellow arrow indicates a hyperintense nodular focus along the floor of the hypothalamus, consistent with an ectopic neurohypophysis - the posterior pituitary tissue, which failed to descend to its normal location within the sella. This structural defect may represent a profound developmental disruption of the oral ectoderm (Rathke’s pouch), the embryological precursor of the anterior pituitary, which we propose links directly to the nuclear factor-kappa B (NF-kB) pathway dysfunction characteristic of incontinentia pigmenti.

Following multidisciplinary evaluation, hormone replacement therapy was initiated at four years and seven months. Given confirmed pituitary aplasia with multiple hormonal deficiencies and markedly suppressed IGF-1, a diagnosis of growth hormone deficiency was established without formal provocative testing, in accordance with current paediatric guidelines [13]. Formal growth hormone stimulation testing was not performed, consistent with current guidelines indicating that a diagnosis of growth hormone deficiency may be established without provocative testing when auxological criteria, a structural hypothalamic-pituitary defect, and deficiency of at least one additional pituitary hormone are all present [13] - all three of which were met in this patient.

The patient began recombinant human growth hormone (rhGH) therapy (daily subcutaneous administration at 20-25.6 µg/kg/day, with subsequent dose adjustment based on IGF-1 response and growth velocity) and hydrocortisone (oral tablets, 9-11 mg/m²/day) to address secondary adrenal insufficiency based on suppressed ACTH and low basal cortisol, both initiated at four years and seven months. Desmopressin (oral tablets, 60-120 µg/day) was initiated separately, at five years and six months, once diurnal enuresis was recognized as clinically significant by the patient’s caregivers, with subsequent symptomatic improvement. This delayed recognition further illustrates how subtle endocrine symptoms in IP may go unnoticed until they become more pronounced or until caregiver awareness increases, reinforcing the importance of proactively screening for endocrine dysfunction.

In a three-year period of follow-up, treatment resulted in an adequate growth response, with serum IGF-1 values normalizing to 262 ng/mL (reference values 87.3 - 324 ng/mL). No adverse effects related to rhGH therapy were observed, nor any worsening of cutaneous lesions noted.

Clinical evolution showed a marked improvement in growth velocity, following initiation of rhGH therapy. Height z-score improved progressively (from -2.32 SDS to -1.58 SDS), accompanied by a gradual increase in Body Mass Index (-0.17 SDS to +1.1 SDS) (Figure 3).

**Figure 3:**
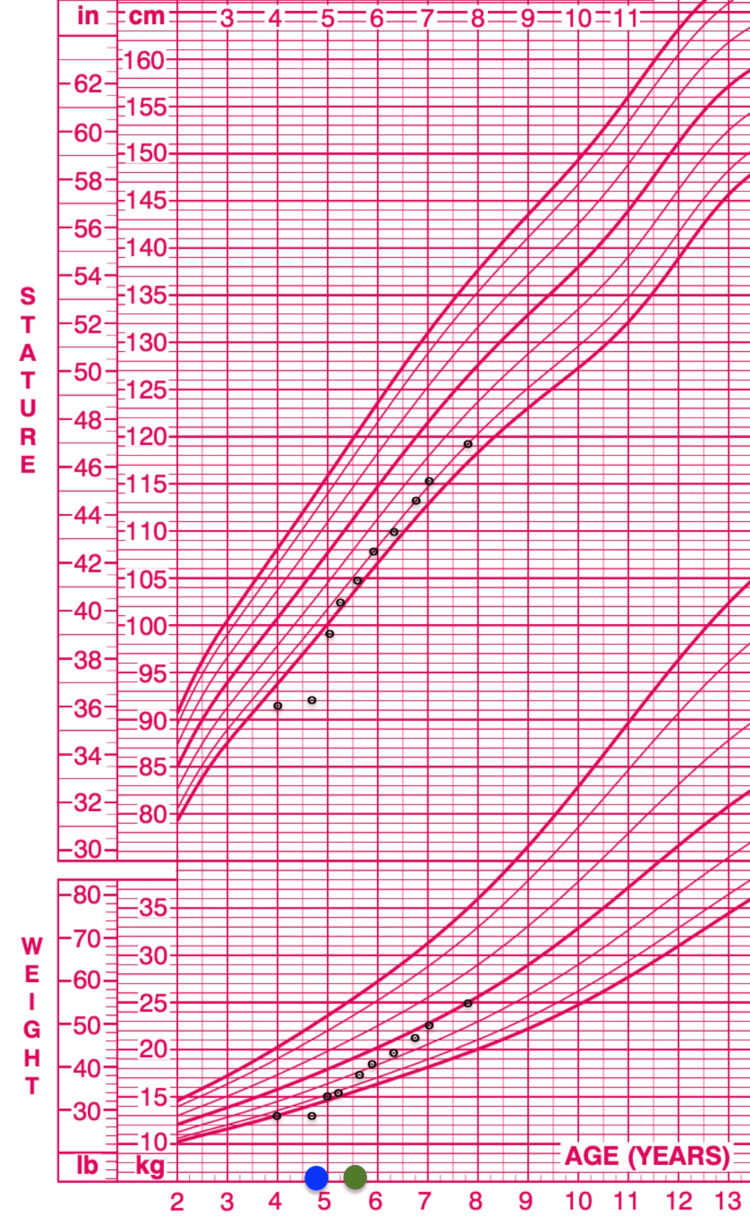
Growth chart showing height and weight progression The blue circle marks the initiation of recombinant human growth hormone (rhGH) and hydrocortisone (age four years seven months); the green circle marks the initiation of desmopressin (age five years six months). Adapted from the public domain CDC Growth Charts: United States [14].

Currently, being seven years and seven months old, the patient demonstrates appropriate neuropsychomotor development. Gross and fine motor skills are preserved, including normal gait, balance, and coordination, with appropriate manual dexterity for daily tasks. Language development is within normal limits, with age-appropriate expressive and receptive communication skills. Cognitive function appears preserved, as evidenced by adequate learning abilities, attention span, and school performance consistent with her age group. Social and adaptive behaviors are also appropriate, with normal interaction patterns and independence in daily activities. Ophthalmological evaluations remain normal. Neurological examination is unremarkable. There is no history of seizures, developmental regression, or behavioral abnormalities. She remains under multidisciplinary follow-up including stomatological follow-up for hypoplasia affecting the molar teeth of the left maxillary arch.

## Discussion

IP is a rare X-linked dominant neurocutaneous ectodermal disorder. It has a high penetrance, with most patients expressing the phenotype within a few months after birth [8].

IP mainly occurs in females, as it is usually fatal in utero for males. However, some males survive due to 47, XXY karyotype, somatic mosaicism or hypomorphic mutations [1,2,15]. In the present case, the previous family history of a medically terminated pregnancy due to nonspecific polymalformative syndrome with a 46,XY karyotype is consistent with what is described in the literature regarding IP-related male fetal lethality. 

Being an ectodermal disorder, skin involvement occurs in almost all patients. IP may also involve teeth, eyes, hair, central nervous system, bone structures, skeletal musculature and immune system [15].

The suspicion of IP is based on major and minor criteria. The major criteria are the typical skin lesions that progress through four stages from infancy to adulthood: vesicular lesions, verrucous lesions, hyperpigmented lesions along Blaschko lines, and hypopigmentated lesions, during adulthood. More than one type of lesion may be present at any time, and its locations can vary from stage to stage [15,16].

Among minor criteria are teeth, hair, nails and retina abnormalities, as well as family history consistent with X-linked inheritance or a history of multiple miscarriages. The presence of minor criteria supports the clinical diagnosis, with some authors suggesting that central nervous system, palate, and breast anomalies be added to the minor diagnostic criteria [15,16].

The diagnosis of IP is established if at least one major criterion is present. If clinical features are inconclusive, the diagnosis of IP can be established after molecular genetic testing [8].

In this case, the patient presented with both major and minor criteria for IP: typical vesicular skin lesions, corresponding to the first stage, as well as craniofacial dysmorphisms (narrow palate). The diagnosis was made according to the clinical presentation and confirmed by the genetic testing. Cutaneous lesions resolved just with topical corticosteroids without any further complications [16,17]. Usually, the cutaneous lesions of IP resolve on their own without any specific treatment. In the first stages, topical anti-inflammatory medications, emollients and standard measures to reduce the risk of skin infection can be used to avoid secondary bacterial infections [15,16].

Regarding neurological involvement, 30% of individuals with IP have seizures, intellectual disability, spastic paresis and brain anomalies. While primary brain anomalies are rare, the literature reports cases of agenesis of the corpus callosum with an occipital encephalocele, polymicrogyria, and gray matter heterotopias. Periventricular leukomalacia was identified on brain MRI in 27 of 43 individuals with IP who have neurocognitive disabilities [8].

While IP involves multiple systems, endocrine involvement remains rarely reported and poorly characterized. To our knowledge, this represents the first documented case of complete pituitary aplasia in a patient with genetically confirmed IP; the three-year follow-up under rhGH therapy further provides novel longitudinal data on the safety and efficacy of this treatment in the context of IP.

IP is caused by mutations in *NEMO/IKBKG*, which impair NF-kB signalling. NF-kB normally induces anti-apoptotic gene transcription, and its dysfunction in IP leaves ectodermal cells vulnerable to apoptotic stimuli such as tumor necrosis factor α (TNFα) [5] - the basis for the skin, dental, and ocular findings classically seen in this disorder. The anterior pituitary is also ectodermal in origin, arising from the oral ectoderm via Rathke’s pouch. We therefore hypothesize that the same NF-kB-mediated apoptotic vulnerability that affects other ectodermal tissues in IP may similarly disrupt pituitary progenitor cells during Rathke’s pouch development, contributing to the pituitary aplasia observed in this case. Supporting this plausibility, a related NF-kB family member, *NFKB2*, is required for normal pituitary corticotroph differentiation [17], and NF-kB signalling more broadly has established roles in the development of other ectoderm-derived organs, such as the liver, skin, and lung [18]. However, this specific role for *NEMO/IKBKG* in pituitary organogenesis remains hypothetical.

The phenotypic variability of IP is largely attributed to functional X-chromosome mosaicism: cells expressing the mutant *IKBKG* allele are progressively eliminated through selective apoptosis, resulting in skewed X-inactivation favoring cells expressing the wild-type allele [5,8]. This stochastic, tissue-specific mosaicism - well established as the basis for the variable penetrance of CNS, dental, and ocular involvement in IP - may similarly explain why pituitary involvement is not a universal feature, despite the shared underlying NF-kB pathway defect.

This case highlights the importance of systematic endocrine evaluation in IP patients presenting with growth failure or clinical signs of hormonal deficiencies. Further studies are needed to better elucidate the spectrum of hypothalamic-pituitary involvement in IP and to establish evidence-based screening recommendations for endocrine complications in this rare disorder. Notably, while growth failure was readily identified through routine percentile-based screening, diurnal enuresis - a more subtle and nonspecific sign - was initially considered within the differential of neurodevelopmental or behavioral causes before being recognized as a manifestation of central diabetes insipidus. This illustrates the variable ways in which pituitary hormone deficiencies may present in children with IP, and underscores the importance of keeping endocrine dysfunction in the differential diagnosis even when symptoms appear nonspecific or overlap with neurodevelopmental concerns. It also demonstrates the safety and efficacy of rhGH therapy in a patient with IP over a three-year follow-up, with no exacerbation of dermatological manifestations. This three-year safety signal is, in our view, one of the most clinically valuable findings of this report. IGF-1 - the principal mediator of rhGH action - is a recognized stimulator of keratinocyte proliferation [19], raising a theoretical concern that rhGH therapy could aggravate the abnormal keratinocyte turnover characteristic of IP. The absence of cutaneous worsening over three years of treatment suggests that systemic IGF-1 elevation does not necessarily reactivate disease activity in healed or quiescent IP lesions, although this observation is based on a single patient and would benefit from confirmation in additional cases.

Those patients must have a multidisciplinary team follow-up with regular examinations for dental, CNS, and ophthalmic abnormalities [15,16]. In the absence of substantial neonatal or infantile complications, patients should have normal life expectancy and a favorable prognosis [8].

## Conclusions

This case describes, to our knowledge, the first documented association between genetically confirmed IP and complete pituitary aplasia. The diagnosis was established through a combination of clinical, biochemical, and neuroimaging findings, and hormone replacement therapy - including rhGH - resulted in significant clinical improvement without exacerbation of cutaneous manifestations over a three-year follow-up.

This case highlights the importance of endocrine evaluation in IP patients presenting with growth failure or other clinical signs of hormonal deficiency. Given the single-case nature of this report, we suggest that a low threshold for pituitary function assessment may be warranted in this population when such signs are present. This rationale follows from the fact that the anterior pituitary arises from the oral ectoderm via Rathke’s pouch, and may therefore be similarly vulnerable to the NF-kB pathway dysfunction caused by NEMO/IKBKG mutations during this developmental process - a mechanism discussed in detail above. Broader generalization to asymptomatic patients would require further study.
